# Efficacy of PD-1/PD-L1 inhibitors combined with anti-VEGF/TKIs and TACE in uHCC: a meta-analysis

**DOI:** 10.3389/fonc.2026.1927223

**Published:** 2026-09-14

**Authors:** Xiaohong Kang, Shiyan Zhang, Qiufeng Zhang, Zeqi Tang, Jiani Ji, Minyuan Ni, Li Wang

**Affiliations:** 1 Department of General Surgery, Ningbo Hospital of Integrated Traditional Chinese and Western Medicine, Ningbo, Zhejiang, China; 2 The Second Clinical Medical College of Zhejiang Chinese Medical University, Zhejiang Chinese Medical University, Hangzhou, Zhejiang, China; 3 The Fifth School of Clinical Medical, Zhejiang Chinese Medical University, Hangzhou, Zhejiang, China

**Keywords:** hepatocellular carcinoma, meta-analysis, PD-1/PD-L1 inhibitors, prognosis, targeted therapy, transarterial chemoembolization

## Abstract

**Background:**

This study evaluated the efficacy of transarterial chemoembolization (TACE) combined with anti-vascular endothelial growth factor (VEGF)/tyrosine kinase inhibitors (TKIs) and programmed cell death protein 1 (PD-1)/programmed cell death protein ligand 1 (PD-L1) inhibitors in the treatment of unresectable hepatocellular carcinoma (uHCC).

**Methods:**

Four databases were searched for studies evaluating the efficacy of TACE combined with anti-VEGF/TKIs and PD-1/PD-L1 inhibitors in uHCC. Pooled data were analyzed using random-effects models, with hazard ratio (HR) and 95% confidence intervals reported. Overall survival (OS) was the primary outcome, and progression-free survival (PFS) was a secondary outcome. Subgroup analyses were performed based on treatment regimens, therapy setting, and the sequencing of TACE and systemic therapy.

**Results:**

A total of 53 studies (3 multicentre randomized controlled trials (RCTs) and 50 cohort studies) comprising over 10, 000 patients were included. Patients receiving triple therapy may derive greater OS (HR = 0.47, p<0.001) and PFS (HR = 0.52, p<0.001) benefits than those receiving other regimens. In OS subgroups, across various combinations, the TACE-bevacizumab-atezolizumab (HR = 0.44, p<0.001) and TACE-lenvatinib-tislelizumab regimens (HR = 0.44, p<0.001) appear to derive greater benefit. In contrast, the TACE-lenvatinib-pembrolizumab combination appears to show less benefit (HR = 0.67, p<0.001). Additionally, patients could potentially carry greater OS benefit with triple therapy, regardless of whether TACE was performed first (HR = 0.46, p<0.001) or systemic therapy was followed by TACE (HR = 0.42, p=0.001). Regarding treatment setting, the first-line group may obtain greater OS benefit (HR = 0.47, p<0.001), and the non-first-line group may also derive greater benefit (HR = 0.34, p=0.001).

**Conclusion:**

Triple therapy (TACE with anti-VEGF/TKIs and PD-1/PD-L1 inhibitors) could significantly improve prognosis in uHCC, and its efficacy may vary depending on the treatment regimen, including the drug combination, treatment sequence, and lines.

**Systematic Review Registration:**

https://www.crd.york.ac.uk/PROSPERO/view/CRD420251151703, identifier CRD420251151703.

## Introduction

In 2022, liver cancer ranked as the sixth most commonly diagnosed cancer and the third leading cause of cancer-related deaths worldwide. It accounted for 7.8% of all cancer-related deaths globally (1). The predominant risk factor for hepatocellular carcinoma (HCC) was cirrhosis, usually caused by hepatitis B virus (HBV) or hepatitis C virus infection, alcohol-related liver disease, or metabolic dysfunction-associated steatotic liver disease (2). Once diagnosed with HCC, 80% of cases were typically at an advanced stage, making surgical resection impossible (3). As a result, unresectable hepatocellular carcinoma (uHCC) still accounted for most newly diagnosed HCC cases and has a poor prognosis.

Transarterial chemoembolization (TACE) is widely recognized as the standard of care for patients with intermediate to advanced-stage HCC. Subsequently, the discovery of sorafenib (Sor) heralded the advent of the era of targeted therapy in HCC. Following the era of targeted therapy, the introduction of immune checkpoint inhibitors (ICIs), either as monotherapy or in combination with targeted agents, has yielded significant improvements in patient outcomes. For instance, the milestone IMbrave150 trial (4) showed that the combination of atezolizumab (Ate) and bevacizumab (Bev) provided a greater survival benefit than Sor alone. Currently, triple therapy combining TACE with anti-vascular endothelial growth factor (VEGF)/tyrosine kinase inhibitors (TKIs) and programmed cell death protein 1 (PD-1)/programmed cell death protein ligand 1 inhibitors (PD-L1) inhibitors is considered among the most promising treatment strategies for uHCC, as exemplified by the following phase III randomized controlled trials (RCTs). The LEAP-012 trial (NCT04246177) (5) evaluated TACE in combination with lenvatinib (Len) plus pembrolizumab (Pem) versus TACE alone, enhancing the 24-month overall survival (OS) rate from 69% to 75%. Additionally, the EMERALD-1 trial (NCT03778957) (6) assessed TACE plus durvalumab (Dur) with Bev. It demonstrated that the triple therapy reduced the risk of disease progression or death by 23% (hazard ratio (HR)=0.77) compared with TACE alone. Although both of the aforementioned RCTs have demonstrated the efficacy of triple therapy, the optimal treatment regimen for uHCC remains unclear.

Recently, a network meta-analysis (7) demonstrated that Ate plus Bev achieved the optimal benefit in progression-free survival (PFS) among systemic therapies for advanced HCC with Child-Pugh B cirrhosis. By systematically reviewing large-scale RCTs and related cohort studies, our meta-analysis aimed to evaluate the efficacy and optimal management of the triple therapy.

## Methods

### Search strategy

Two researchers independently searched PubMed, Web of Science, Embase, and the Cochrane Library for studies on triple therapy (TACE combined with targeted and immunotherapy) for the treatment of uHCC, published from database inception through August 2026. Additionally, a manual review of additional sources was conducted to ensure no relevant studies were overlooked. The search strategy employed a combination of Medical Subject Headings and free-text terms. These terms included (“hepatocellular carcinoma” OR “HCC”) AND (“transarterial chemoembolization” OR “TACE”) AND (“molecular targeted therapy” OR “TKIs” OR “anti-VEGF” OR “apatinib” OR “bevacizumab” OR “lenvatinib” OR “sorafenib”) AND (“immune checkpoint inhibitors” OR “ICIs” OR “PD-1 inhibitor” OR “PD-L1 inhibitor” OR “atezolizumab” OR “camrelizumab” OR “pembrolizumab” OR “sintilimab” OR “tislelizumab”). Given the wide variety of targeted drugs and PD-1/PD-L1 inhibitors used in clinical practice, the search was not limited to the agents listed above. Adhering to the Preferred Reporting Items for Systematic Reviews and Meta-Analyses guidelines, this study was registered with PROSPERO (CRD420251151703).

### Inclusion and exclusion criteria

Two researchers independently selected the publications, and disagreements were resolved through discussion. Articles were considered for inclusion if they met the following criteria: (1) patients suffered from uHCC; (2) the experimental group received triple therapy (TACE combined with anti-VEGF/TKIs and PD-1/PD-L1 inhibitors); (3) the control group received any dual therapy or monotherapy (e.g., TACE alone, TACE plus targeted therapy, TACE plus PD-1/PD-L1 inhibitors, or targeted therapy plus PD-1/PD-L1 inhibitors); (4) cohort studies or RCTs; (5) studies reported outcomes in terms of OS or PFS; (6) the total number of patients in the study was at least 100, with more than 30 cases in each group.

Studies were excluded if they met any of the following criteria: (1) unavailability of full texts; (2) absence of available data; (3) updated studies; (4) including patients with resectable HCC; (5) patients received a combination of treatments, such as hepatic arterial infusion chemotherapy, radiotherapy, radiofrequency ablation, or surgical resection.

### Outcome measures

In this study, the primary outcome was OS, defined as the interval from the start of follow-up to death from any cause or the most recent follow-up. The secondary outcome was PFS, measured from treatment initiation until the first occurrence of tumor progression, death from any cause, or the last follow-up.

### Data extraction

Data extraction was independently performed by two investigators using a standardized data extraction form based on predefined inclusion and exclusion criteria. In the event of discrepancies, resolution was achieved through consensus discussion. Extracted information encompassed: (1) study characteristics such as authors, publication year, country, study design, recruitment time, treatment regimen, and sample size; (2) patient demographics, including age, sex, the score of Eastern Cooperative Oncology Group performance status (ECOG PS), Barcelona Clinic Liver Cancer (BCLC) stage, and Child-Pugh class; (3) survival outcomes.

### Quality assessment

The quality of the included RCTs was evaluated using the Cochrane Collaboration’s Risk of Bias Tool, which included criteria such as random sequence generation, allocation concealment, blinding of participants and personnel, blinding of outcome assessment, incomplete outcome data, selective reporting, and other potential biases. Each criterion was categorized as presenting a high, low, or unclear risk. To assess the quality of cohort studies, the Newcastle-Ottawa Scale (NOS) was employed, comprising three dimensions: selection, comparability, and outcomes. In this study, studies with scores of 6 or higher were considered high quality. The overall certainty of evidence for OS and PFS was assessed using the GRADE (Grading of Recommendations Assessment, Development and Evaluation) methodology in GRADEpro, based on the five GRADE domains (risk of bias, inconsistency, indirectness, imprecision, and publication bias).

### Statistical analysis

This meta-analysis was conducted in Stata (version 12.0), except for the sensitivity analysis, which was performed in R (version 4.5.3). Treatment effects were measured using the HR and its corresponding 95% confidence interval (95%CI). Given potential heterogeneity among the included studies, including differences in study design, participant demographics, and treatment regimens, a random-effects model was employed to yield more reliable statistical outcomes. Results favoring the experimental group were indicated by an HR<1, whereas an HR>1 indicated greater benefit in the control group. Heterogeneity among studies was assessed using the chi-square (χ²) test and quantified by the I² statistic, with I²<25% indicating no heterogeneity, I² in the range of 25 to 50% indicating low heterogeneity, I² in the range of 50 to 75% indicating moderate heterogeneity, and I²>75% indicating high heterogeneity. Subgroup analyses were performed based on treatment regimen, timing, and therapy setting. Additionally, sensitivity analyses were conducted to identify potential sources of heterogeneity and to assess the robustness of the findings. These included omitting individual studies one at a time and comparing the results of random-effects and fixed-effects models. Publication bias was assessed using Egger’s test. All statistical tests were two-sided, with a significance threshold set at p<0.05.

## Results

### Study selection

Using predefined search terms, 2518 records were identified through an initial comprehensive search of PubMed, Web of Science, the Cochrane Library, and Embase. After a manual review, 2 additional records from other sources were included. After removing duplicates, 952 records remained, while 847 records were excluded. After careful examination of titles and abstracts, eligibility assessments were conducted for 105 studies. After full-text evaluation, 52 studies were excluded for various reasons, including limited sample size (n = 33), updated studies (n = 13), and data unavailable (n = 6); a complete list of these excluded studies and reasons was provided in **Supplementary Material**. As a result, 53 studies (5, 6, 8–58) were included in the final analysis. A detailed overview of the literature selection procedure was presented in **Figure 1**.

**Figure 1:**
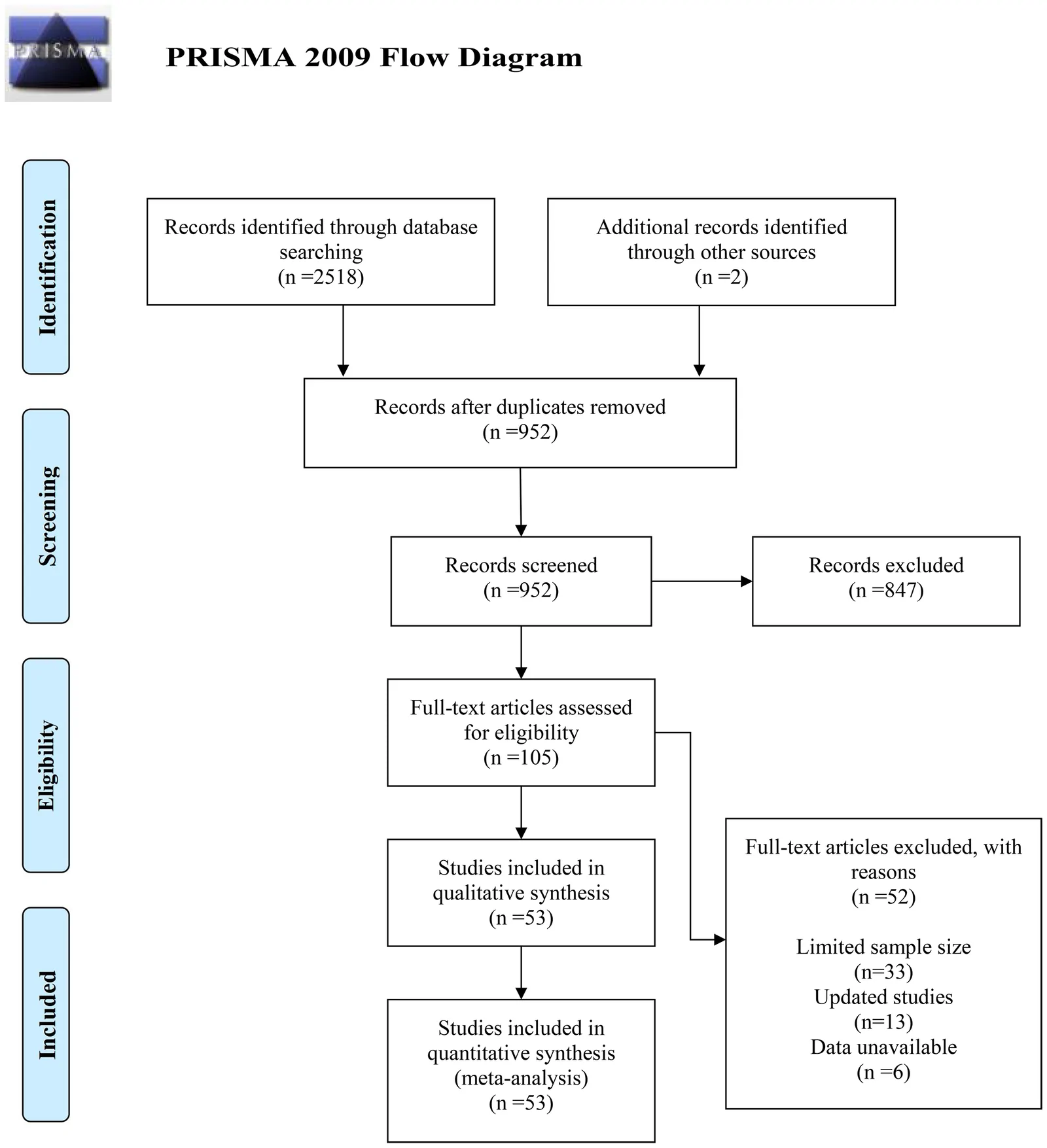
Flow chart of study selection.

### Study characteristics

Published between 2022 and 2026, the studies included in this analysis encompassed over 10, 000 uHCC patients. Among these studies, three were multicentre RCTs, while 50 were cohort studies. Of these cohort studies, forty-nine were conducted in China and 1 in Japan. The experimental group comprised more than 5, 700 patients, whereas the control group comprised more than 5, 500 patients. Various targeted drugs were utilized across the included studies, involving Bev (its biosimilar) and TKIs such as Len, Sor, Bev, apatinib (Apa), donafenib, and regorafenib. Additionally, a wide range of PD-1/PD-L1 inhibitors were employed, including Ate, Dur, Pem, camrelizumab (Cam), sintilimab (Sin), tislelizumab (Tis), and toripalimab (Tor). Detailed characteristics of the studies were summarized in **Table 1**. Furthermore, additional details on the included literature, including BCLC stage, Child-Pugh class, and ECOG PS, can be found in **Supplementary Table 1**.

**Table 1:** Basic characteristics of included studies.

Author	Year	Intervene		Control		Anti-VEGF/TKIs	PD-1/PD-L1 inhibitors	Primary outcomes
		Sample size	Regimen	Sample size	Regimen			
Kudo M (5)	2025	237	TACE+Len+Pem	243	TACE+PBO	Len	Pem	OS, PFS
Sangro B (6)	2025	204	TACE+Bev+Dur	205	TACE+PBO	Bev	Dur	PFS
Zhu HD (8)	2026	100	TACE+Apa+Cam	100	TACE	Apa	Cam	OS, PFS
Yang D-L	2025	301	TACE+Bev/TKIs+PD-1/PD-L1 inhibitors	301	TACE	Bev/TKIs	PD-1/PD-L1 inhibitors	OS, PFS
Yao Y (10)	2025	39	TACE+Len+PD-1/PD-L1 inhibitors	39	TACE	Len	Cam/Tis	OS
Wang M-X (11)	2025	122	TACE+TKIs+PD-1/PD-L1 inhibitors	52	TACE+TKIs	Len/Sor/Don	Cam/Sin	OS, PFS
Zheng Y (12)	2024	42	TACE+Bev+Ate	42	TACE	Bev	Ate	OS, PFS
Guo C (13)	2025	26	TACE+Bev/TKIs+PD-1/PD-L1 inhibitors	26	TACE	Bev/TKIs	PD-1/PD-L1 inhibitors	OS, PFS
Yuan G (14)	2024	139	TACE+TKIs+PD-1/PD-L1 inhibitors	278	TACE	Len/Sor/Apa/Reg	PD-1/PD-L1 inhibitors	OS
Tang Z (15)	2024	70	TACE+Len+Cam	70	TACE	Len	Cam	OS, PFS
Jin ZC (16)	2024	805	TACE+Anti-VEGF/TKIs+PD-1/PD-L1 inhibitors	437	TKIs+PD-1/PD-L1 inhibitors	Anti-VEGF/TKIs	PD-1/PD-L1 inhibitors	OS, PFS
Jiang X	2024	44	TACE+TKIs+Cam	83	TACE+TKIs	Len/Sor	Cam	OS, PFS
Jiang J (18)	2024	68	TACE+Len+Tis	68	TACE+Len	Len	Tis	OS, PFS
Chen S (19)	2024	66	TACE+Len+Tis	45/38	TACE+Len/TACE	Len	Tis	OS, PFS
Cao L	2025	56	TACE+Reg+PD-1/PD-L1 inhibitors	56	Reg+PD-1/PD-L1 inhibitors	Reg	Cam/Sin	OS, PFS
Yang H (21)	2023	64	TACE+Len+PD-1/PD-L1 inhibitors	58	TACE+Len	Len	Cam/Tis/Tor	PFS
Wu J (22)	2023	57	TACE+Len+Cam	41/43	TACE+Cam/TACE	Len	Cam	OS, PFS
Wang J (23)	2023	43	TACE+Len+PD-1/PD-L1 inhibitors	43	Len+PD-1/PD-L1 inhibitors	Len	Cam/Sin/Pem	OS, PFS
Sun T	2023	53	TACE+TKIs+Cam	53	TACE+TKIs	Len/Sor/Apa	Cam	OS, PFS
Li H (25)	2023	166	TACE+Don+PD-1/PD-L1 inhibitors	157	TACE+Don	Don	Cam/Tis/Tor	OS, PFS
Lang M (26)	2023	75	TACE+Len+Sin	39	Len+Sin	Len	Sin	OS, PFS
Gao Y (27)	2023	57	TACE+TKIs+Cam	50	TACE+TKIs	Len/Sor	Cam	OS, PFS
Duan X (28)	2023	449	TACE+Apa+Cam	449	TACE+Apa	Apa	Cam	OS, PFS
Zhu D (29)	2022	34	TACE+Apa+Cam	68	TACE+Apa	Apa	Cam	OS
Xiang YJ (30)	2022	56	TACE+Len+PD-1/PD-L1 inhibitors	47	TACE+PD-1/PD-L1 inhibitors	Len	Tor/Sin	OS, PFS
Qu S (31)	2022	56	TACE+Len+PD-1/PD-L1 inhibitors	54	TACE	Len	Tor/Pem	OS, PFS
Zou X (32)	2023	70	TACE+Len+PD-1/PD-L1 inhibitors	90	TACE+Len	Len	Pem/Sin	OS, PFS
Hong X (33)	2024	77	TACE+Bev/TKIs+PD-1/PD-L1 inhibitors	62	TKIs+PD-1/PD-L1 inhibitors	Bev/Len/Sor	Cam/Sin	OS, PFS
Gao B (34)	2023	37	TACE+TKIs+PD-1/PD-L1 inhibitors	37	TACE	Len/Sor	Cam/Sin	OS, PFS
Cao F (35)	2023	62	TACE+Bev+Ate	77	Bev+Ate	Bev	Ate	OS, PFS
Li H (36)	2023	46	TACE+TKIs+PD-1/PD-L1 inhibitors	46	TKIs+PD-1/PD-L1 inhibitors	NR	NR	OS, PFS
Sheng Y (37)	2024	113	TACE+Len+PD-1/PD-L1 inhibitors	113	TACE+Len	Len	Cam/Sin/Tis	OS, PFS
Jin M (38)	2024	39	TACE+Len+PD-1/PD-L1 inhibitors	39	Len+PD-1/PD-L1 inhibitors	Len	Cam/Sin/Tis	OS, PFS
Zhao Y (39)	2024	103	TACE+Len+Tis	66	TACE+Len	Len	Tis	OS, PFS
Xia WL (40)	2022	59	TACE+Apa+PD-1/PD-L1 inhibitors	59	TACE+Apa	Apa	Cam/Sin/Tis/Pem	OS
Chen S (41)	2022	70	TACE+Len+Pem	72	TACE+Len	Len	Pem	OS, PFS
Pan H (42)	2025	60	TACE+TKIs+PD-1/PD-L1 inhibitors	60	TKIs+PD-1/PD-L1 inhibitors	Len/Sor	NR	OS, PFS
Han Z (43)	2022	50	TACE+TKIs+PD-1/PD-L1 inhibitors	76/45	TACE+TKIs/TACE	Len/Sor/Apa/Reg	Cam/Sin/Tis/Pem	OS, PFS
Xue M (44)	2025	35	TACE+Len+PD-1/PD-L1 inhibitors	80	TACE+Len	Len	Cam/Sin/Tis/Pem	OS, PFS
Liu S (45)	2025	42	TACE+Len+PD-1/PD-L1 inhibitors	42	TACE+Len	Len	Cam/Sin/Tis	OS, PFS
Liu C-Y (46)	2025	54	TACE+Bev/TKIs+PD-1/PD-L1 inhibitors	57	TKIs+PD-1/PD-L1 inhibitors	Bev/TKIs	PD-1/PD-L1 inhibitors	OS, PFS
Lei Y (47)	2025	210	TACE+Bev/TKIs+PD-1/PD-L1 inhibitors	76	TKIs+PD-1/PD-L1 inhibitors	Bev/Apa/Don/Len	Cam/Sin/Tis/Ate	OS, PFS
Zhang N (48)	2025	158	TACE+Bev+Ate	155	Bev+Ate	Bev	Ate	OS, PFS
Zhu R (49)	2025	83	TACE+Apa+Cam	84	Apa+Cam	Apa	Cam	OS, PFS
Zhang Q (50)	2026	58	TACE +Don+Cam	58	TACE+Don	Don	Cam	OS, PFS
Yang ZR (51)	2025	134	TACE+Len+Pem	126	TACE	Len	Pem	OS, PFS
Wang D (52)	2026	46	TACE+Reg+PD-1/PD-L1 inhibitors	46	Reg+PD-1/PD-L1 inhibitors	Reg	Cam/Sin	OS, PFS
Chen JJ (53)	2026	307	TACE+Anti-VEGF/TKIs+PD-1/PD-L1 inhibitors	633	TACE	Anti-VEGF/TKIs	PD-1/PD-L1 inhibitors	OS, PFS
Zhao H (54)	2026	113	TACE+Len+PD-1/PD-L1 inhibitors	96	TACE+Len	Len	PD-1/PD-L1 inhibitors	OS, PFS
Liang Y	2025	132	TACE+Len+PD-1/PD-L1 inhibitors	132	TACE+Len	Len	PD-1/PD-L1 inhibitors	OS, PFS
Li Z (56)	2026	41	TACE+Bev/TKIs+PD-1/PD-L1 inhibitors	101	TACE	Bev/Len/Sor/Don	PD-1/PD-L1 inhibitors	OS, PFS
Hashimoto K (57)	2025	49	TACE+Bev+Ate	49	Bev+Ate	Bev	Ate	OS, PFS
Xu HW (58)	2025	105	TACE+Anl+Sin	105	TACE+Anl	Anl	Sin	OS, PFS

TACE, transarterial chemoembolization; TKIs, tyrosine kinase inhibitors;VEGF, vascular endothelial growth factor; PD-1, programmed cell death protein 1; PD-L1, programmed cell death protein ligand 1; PBO, placebo; Len, lenvatinib; Bev, bevacizumab; Sor, sorafenib; Don, donafenib; Reg, regorafenib; Apa, apatinib; Anl, anlotinib; Pem, pembrolizumab; Dur, durvalumab; Cam, camrelizumab; Tis, tislelizumab; Sin, sintilimab; Ate, atezolizumab; Tor, toripalimab; PFS, progression-free survival; OS, overall survival; NR, not reported.

### Quality assessment

For RCTs included in this meta-analysis, the Cochrane Collaboration’s Risk of Bias Tool was employed to evaluate their quality. All three RCTs were assessed as having a “low risk” of bias across randomization sequence generation, performance bias, detection bias, attrition bias, and reporting bias. For allocation concealment, two studies were classified as “low risk”, while the remaining study was deemed “high risk” (8). For the domain of other biases, however, insufficient information across all these studies resulted in an “unclear risk” classification. Detailed quality assessments of these studies were summarized in **Supplementary Figures 1** and **2**. NOS was used to assess the quality of cohort studies. Notably, all included studies scored≥6, indicating excellent quality, with some achieving the highest possible NOS score of 9 (36, 42, 52, 55). The specific quality assessment results were detailed in **Supplementary Table 2**. The GRADE assessment rated the certainty of evidence as moderate for both OS and PFS, primarily due to the predominance of cohort studies and substantial statistical heterogeneity. However, the large magnitude of effect (HR<0.5 for both outcomes) upgraded the certainty by one level, with full details available in **Supplementary Table 3**.

### Efficacy-OS

Fifty-one studies reported OS data, indicating that patients treated with triple therapy may derive a greater OS benefit than those in the control group (HR = 0.47, 95%CI[0.43, 0.53], p<0.001), with moderate heterogeneity observed (I²=69.8%), as illustrated in **Figure 2**.

**Figure 2:**
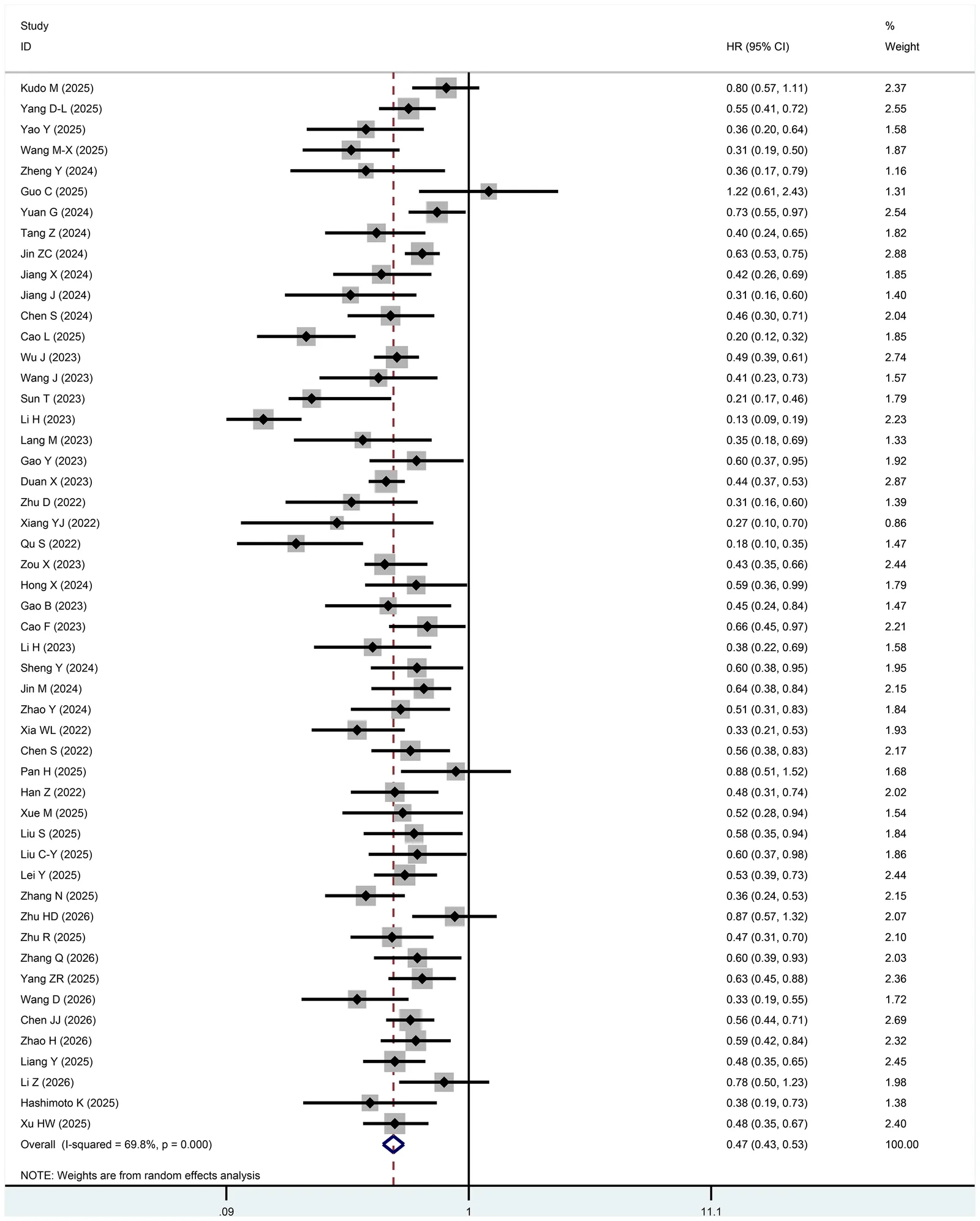
Forest plot showing the effect of PD-1/PD-L1 inhibitors combined with anti-VEGF/TKIs and TACE on OS in patients with uHCC (p<0.001).

### Publication bias and sensitivity analysis for OS

Publication bias was assessed using Egger’s test, suggesting no significant publication bias was observed (p=0.056), as shown in **Supplementary Figure 3**. A sensitivity analysis was conducted by removing studies one at a time, and the results remained stable, as depicted in **Supplementary Figure 4**. This stability was further supported by the fixed-effects model, which yielded point estimates similar to those from the random-effects analysis.

### Subgroup analysis for OS

Subgroup analysis stratified by control treatment showed that patients underwent the triple therapy may carry greater OS benefit compared to those treated with other treatment regimens (TACE alone, HR = 0.54, 95%CI[0.45, 0.64], p<0.001; TACE-TKI, HR = 0.43, 95%CI[0.36, 0.51], p<0.001; TACE-PD-1/PD-L1 inhibitors, HR = 0.43, 95%CI[0.23, 0.81], p=0.010; anti-VEGF/TKI-PD-1/PD-L1 inhibitors, HR = 0.48, 95%CI[0.40, 0.57], p<0.001, respectively). Subgroup analysis was also performed according to specific regimens used in the experimental group. Compared with participants in the control group, patients administered experimental regimens may experience greater OS benefit (TACE-Len-Tis, HR = 0.44, 95%CI[0.33, 0.59], p<0.001; TACE-Bev-Ate, HR = 0.44, 95%CI[0.31, 0.64], p<0.001; TACE-Len-Cam, HR = 0.47, 95%CI[0.39, 0.58], p<0.001; TACE-Apa-Cam, HR = 0.50, 95%CI[0.35, 0.71], p<0.001; TACE-Len-Pem, HR = 0.67, 95%CI[0.54, 0.82], p<0.001, respectively). Among patients in the triple-therapy groups, those in the TACE-Len-Tis and TACE-Bev-Ate groups seem to benefit the most from the experimental treatment compared with the control group, whereas those in the TACE-Len-Pem group appear to benefit the least.

Regarding the timing of TACE, this meta-analysis also conducted a subgroup analysis. Of the 30 studies reporting on patients who underwent TACE prior to systemic therapy in the triple therapy group, the results indicated that patients in the triple therapy group potentially derived greater OS benefit than those in the control group (HR = 0.46, 95%CI[0.40, 0.53], p<0.001). Additionally, only 4 studies initiated systemic therapy first, suggesting that patients who underwent triple therapy may also derive greater benefit than those in the control group (HR = 0.42, 95%CI[0.25, 0.68], p=0.001). Further subgroup analysis by treatment setting showed that 23 studies used triple therapy as first-line treatment, indicating that patients who received triple therapy may carry a greater benefit than those in the control group (HR = 0.47, 95%CI [0.40, 0.55], p<0.001). However, only 3 studies used triple therapy as a non-first-line treatment, and the results suggested that the experimental group may have derived a greater survival benefit than the control group (HR = 0.34, 95%CI[0.18, 0.65], p=0.001). For more details, please refer to **Table 2**.

**Table 2:** Subgroup analysis of overall survival.

Subgroup	No. of studies	HR	95%CI	P	Heterogeneity	
					I^2^	P
Control group						
TACE	16	0.54	(0.45-0.64)	<0.001	65.8%	<0.001
TACE-TKI	21	0.43	(0.36-0.51)	<0.001	71.0%	<0.001
TACE-PD-1/PD-L1 inhibitors	2	0.43	(0.23-0.81)	0.010	35.7%	0.212
Anti-VEGF/TKI-PD-1/PD-L1 inhibitors	15	0.48	(0.40-0.57)	<0.001	61.7%	0.001
Triple therapy regimens						
TACE-Apa-Cam	4	0.50	(0.35-0.71)	<0.001	70.8%	0.016
TACE-Bev-Ate	4	0.44	(0.31-0.64)	<0.001	48.1%	0.123
TACE-Len-Cam	2	0.47	(0.39-0.58)	<0.001	0%	0.466
TACE-Len-Pem	3	0.67	(0.54-0.82)	<0.001	1.2%	0.363
TACE-Len-Tis	3	0.44	(0.33-0.59)	<0.001	0%	0.474
Timing of TACE						
TACE before	30	0.46	(0.40-0.53)	<0.001	69.4%	<0.001
Followed by TKI-PD-1/PD-L1 inhibitors	4	0.42	(0.25-0.68)	0.001	77.0%	0.005
Treatment setting for triple therapy						
First-line therapy	23	0.47	(0.40-0.55)	<0.001	75.5%	<0.001
Non-first-line therapy	3	0.34	(0.18-0.65)	0.001	80.2%	0.006

TACE, transarterial chemoembolization; TKI, tyrosine kinase inhibitor; VEGF, vascular endothelial growth factor; PD-1, programmed cell death protein 1; PD-L1, programmed cell death protein ligand 1; Apa, apatinib; Bev, bevacizumab; Len, lenvatinib; Cam, camrelizumab; Ate, atezolizumab; Pem, pembrolizumab; Tis, tislelizumab; HR, hazard ratio; CI, confidence interval.

### Efficacy-PFS

PFS-related data were reported in 49 studies. Patients who received the combination of TACE and targeted therapy with PD-1/PD-L1 inhibitors appeared to have greater PFS benefit than those who received other treatments (HR = 0.52, 95%CI[0.47, 0.57], p<0.001). Moderate heterogeneity was observed in the results (I²=70.2%), as presented in **Figure 3**.

**Figure 3:**
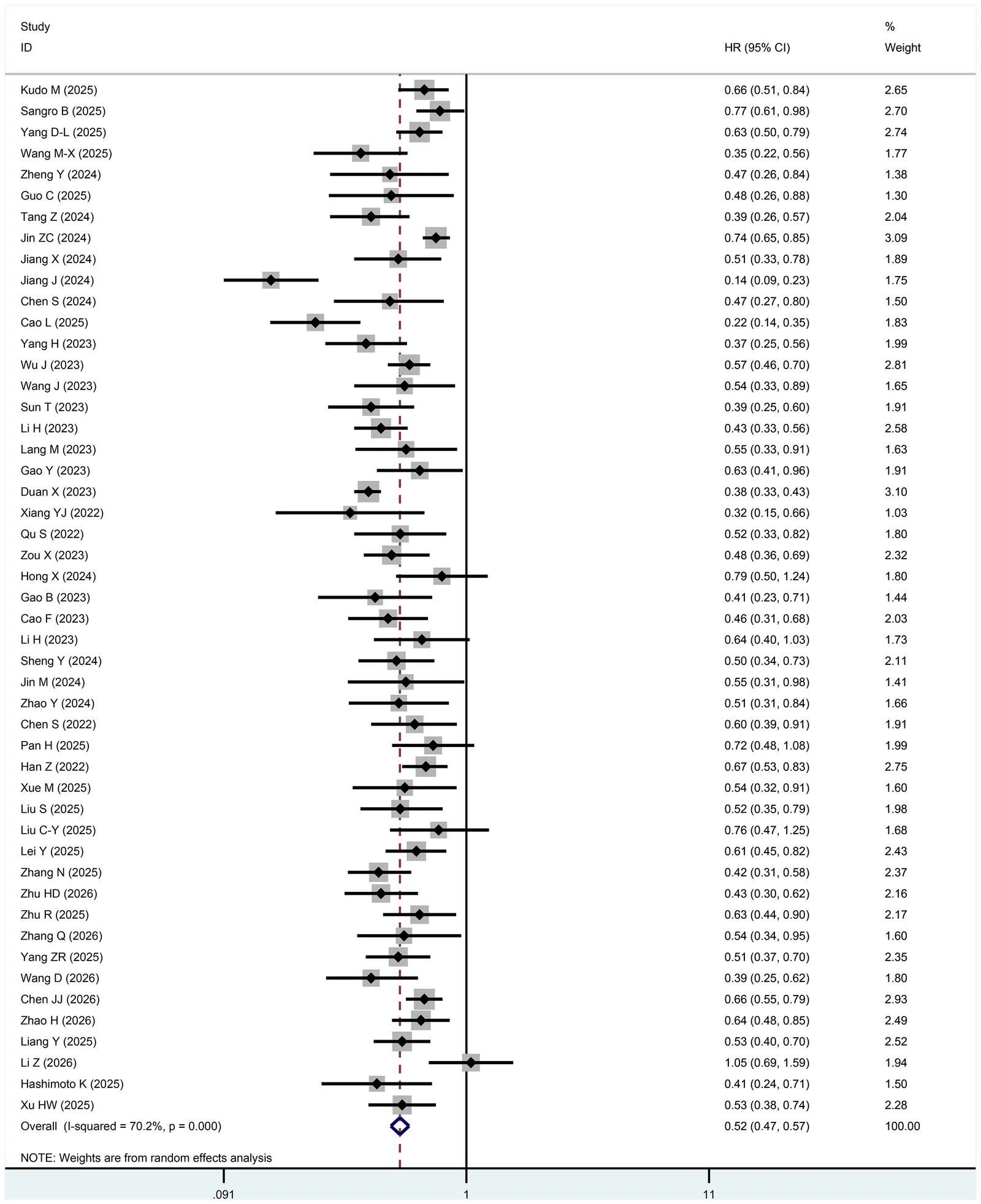
Forest plot showing the effect of PD-1/PD-L1 inhibitors combined with anti-VEGF/TKIs and TACE on PFS in patients with uHCC (p<0.001).

### Publication bias and sensitivity analysis for PFS

Publication bias was evaluated using Egger’s test, which indicated no publication bias (p=0.112), as shown in **Supplementary Figure 5**. The sensitivity analysis showed that PFS results remained stable, as shown in **Supplementary Figure 6**. This stability was further corroborated by fixed-effects modeling, which yielded results consistent with the random-effects analysis.

### Subgroup analysis for PFS

Subgroup analysis based on control treatment demonstrated that patients in the experimental group potentially experienced greater PFS benefit compared to those in the control group (TACE alone, HR = 0.58, 95%CI[0.51, 0.66], p<0.001; TACE-TKI, HR = 0.47, 95%CI[0.41, 0.53], p<0.001; TACE-PD-1/PD-L1 inhibitors, HR = 0.44, 95%CI[0.28, 0.70], p<0.001; anti-VEGF/TKI-PD-1/PD-L1 inhibitors, HR = 0.54, 95%CI[0.46, 0.65], p<0.001). Additional analysis of specific triple therapy regimens indicated that patients in the experimental group may obtain greater PFS benefit than those in the control group (TACE-Len-Tis, HR = 0.32, 95%CI[0.14, 0.74], p=0.008; TACE-Bev-Ate, HR = 0.44, 95%CI[0.35, 0.54], p<0.001; TACE-Len-Cam, HR = 0.49, 95%CI[0.34, 0.70], p<0.001; TACE-Apa-Cam, HR = 0.45, 95%CI[0.34, 0.61], p<0.001; TACE-Len-Pem, HR = 0.60, 95%CI[0.50, 0.72], p<0.001, respectively). Among the various experimental regimens, patients who underwent TACE-Len-Tis appeared to benefit more than those in the control group. In contrast, patients receiving TACE-Len-Pem appeared to derive minimal benefit from triple therapy compared with those in the control group.

The meta-analysis also included a subgroup analysis stratified by the timing of TACE within the triple-therapy framework. In the group in which TACE was administered first, 28 studies contributed to the pooled analysis, indicating a potentially greater PFS benefit for patients treated with triple therapy than for those in the control group (HR = 0.51, 95%CI[0.45, 0.58], p<0.001). In the group in which systemic therapy preceded TACE, 4 studies were analyzed, all demonstrating a potential for greater PFS benefit in patients who underwent triple therapy than in the control group (HR = 0.52, 95%CI[0.42, 0.63], p<0.001). A subgroup analysis by treatment setting found that, in the first-line therapy group, patients receiving triple therapy may derive greater benefit than those in the control group, as reported in 21 studies (HR = 0.52, 95%CI[0.44, 0.61], p<0.001). In contrast, in the non-first-line therapy group, as analyzed in only 3 studies, patients in the experimental group appeared to have greater benefits than those in the control group (HR = 0.38, 95%CI[0.21, 0.69], p=0.001). The corresponding details were presented in **Table 3**.

**Table 3:** Subgroup analysis of progression-free survival.

Subgroup	No. of studies	HR	95%CI	P	Heterogeneity	
					I^2^	P
Control group						
TACE	15	0.58	(0.51-0.66)	<0.001	55.2%	0.005
TACE-TKI	19	0.47	(0.41-0.53)	<0.001	62.5%	<0.001
TACE-PD-1/PD-L1 inhibitors	2	0.44	(0.28-0.70)	<0.001	21.2%	0.260
Anti-VEGF/TKI-PD-1/PD-L1 inhibitors	15	0.54	(0.46-0.65)	<0.001	67.8%	<0.001
Triple therapy regimens						
TACE-Apa-Cam	3	0.45	(0.34-0.61)	<0.001	70.3%	0.034
TACE-Bev-Ate	4	0.44	(0.35-0.54)	<0.001	0%	0.972
TACE-Len-Cam	2	0.49	(0.34-0.70)	<0.001	64.2%	0.095
TACE-Len-Pem	3	0.60	(0.50-0.72)	<0.001	0%	0.459
TACE-Len-Tis	3	0.32	(0.14-0.74)	0.008	87.8%	<0.001
Timing of TACE						
TACE before	28	0.51	(0.45-0.58)	<0.001	70.5%	<0.001
Followed by TKI-PD-1/PD-L1 inhibitors	4	0.52	(0.42-0.63)	<0.001	0%	0.769
Treatment setting for triple therapy						
First-line therapy	21	0.52	(0.44-0.61)	<0.001	81.7%	<0.001
Non-first-line therapy	3	0.38	(0.21-0.69)	0.001	81.5%	0.005

TACE, transarterial chemoembolization; TKI, tyrosine kinase inhibitor; VEGF, vascular endothelial growth factor; PD-1, programmed cell death protein 1; PD-L1, programmed cell death protein ligand 1; Apa, apatinib; Bev, bevacizumab; Len, lenvatinib; Cam, camrelizumab; Ate, atezolizumab; Pem, pembrolizumab; Tis, tislelizumab; HR, hazard ratio; CI, confidence interval.

## Discussion

This updated meta-analysis, comprising 53 studies, evaluated the effectiveness of TACE combined with anti-VEGF/TKIs and PD-1/PD-L1 inhibitors in patients with uHCC, focusing on specific treatment regimens, lines of therapy, and timing. Consistent with previous studies, our results suggest that triple therapy, TACE combined with anti-VEGF/TKIs and PD-1/PD-L1 inhibitors, significantly improved prognosis. For instance, a recently published meta-analysis (59) pooled 12 retrospective studies and found that TACE combined with TKIs and PD-1 inhibitors was associated with superior efficacy compared with TACE plus TKIs. However, this meta-analysis used risk ratios rather than HRs, which limited the strength of the evidence. Another meta-analysis (60) compared TACE plus Len and PD-1 inhibitors with other non-triple therapies and found that triple therapy significantly improved OS and PFS. Nevertheless, this analysis did not further explore the optimal triple-therapy scheme.

With multiple molecular alterations and a multifaceted tumor microenvironment (TME), HCC is highly heterogeneous (61), which may explain the limited efficacy of traditional treatments. Mechanistically, integrating TACE with both targeted therapy and immunotherapy was indispensable. Through intra-arterial chemotherapy and arterial embolization, TACE induces ischemic necrosis and reshapes the TME, thereby treating the tumor locally while creating favorable conditions for subsequent systemic therapies. By the way, articles included in this meta-analysis encompassed two forms of TACE: conventional TACE (cTACE) and drug-eluting bead TACE (DEB-TACE). Compared with cTACE, DEB-TACE contains beads saturated with the chemotherapeutic agent, thereby increasing drug concentration in the tumor and reducing its systemic concentration. Chernyshenko et al. (62) compared DEB-TACE with cTACE, and found that DEB-TACE improved OS by 3.54 months and PFS by 3.07 months compared to cTACE. However, in our study, only a few articles reported exclusively on DEB-TACE, preventing us from performing a subgroup analysis by TACE type.

The rapid growth of HCC demands an abundant supply of nutrients and oxygen. By inhibiting key oncogenic pathways, targeted agents, whether TKIs or anti-VEGF antibodies, suppress tumor cell proliferation and survival. With the concept of vascular normalization proposed by Rakesh K. Jain (63), the management of uHCC entered a new phase. By blocking angiogenesis, targeted drugs help prune abnormal vessels while remodeling the remaining vasculature, thereby enhancing oxygen delivery and improving drug penetration. More and more targeted drugs were being utilized in the management of uHCC. In our study, various targeted agents were included, including TKIs and the anti-VEGF antibody (Bev). Len, an oral receptor TKI, blocks vascular endothelial growth factor receptors (VEGFR) 1-3, fibroblast growth factor receptors (FGFR), platelet-derived growth factor receptor-alpha, and RET and KIT proto-oncogenes, exerting broad anti-angiogenic and immunomodulatory effects (64). Apa, a highly selective VEGFR2 inhibitor that inhibits tumor cell proliferation and angiogenesis, has been proposed as a promising treatment for uHCC (65). Unlike the two therapeutic agents described above, Bev, a macromolecular targeted drug against VEGF-A, is used in cancer treatment to inhibit malignant cell growth and angiogenesis. In conclusion, these targeted agents serve a dual function in the management of uHCC. They not only suppress tumor cell proliferation and promote vascular normalization but also enhance drug delivery, thereby providing ICIs with a more favorable therapeutic environment.

Through antigen presentation machinery and recruitment of immunosuppressive cells, tumors remodel the TME into an immunosuppressive state across many cancer types, providing a rationale for immunotherapy, particularly ICIs targeting T-cell exhaustion pathways, such as PD-1/PD-L1. PD-1, on the other hand, expressed on activated T cells, binds to its ligands, like PD-L1, which is expressed on various cells within the TME, leading to T-cell inhibition. By blocking these targets, immunotherapy can restore and sustain T-cell function, synergizing with TACE and targeted agents to produce durable, systemic anti-tumor effects. In addition to its direct antitumor effect, TACE can reshape the TME by modulating effector T-cell infiltration and reducing immunosuppressive cells, such as regulatory T cells and myeloid-derived suppressor cells (MDSCs), thereby improving the efficacy of subsequent ICIs. Additionally, by promoting the release of tumor-associated antigens, TACE facilitates T-cell activation and proliferation, thereby creating a pre-existing state of immune activation that favors sequential immune therapy (66). Furthermore, TACE has been reported to induce epigenetic changes in HCC (67). Specifically, by inhibiting DNA methylation, TACE can upregulate immune checkpoint expression, such as PD-L1, potentially enhancing tumor immunogenicity and rendering tumors more responsive to ICIs. Similarly, by inhibiting angiogenesis, targeted agents promote vascular normalization, thereby alleviating hypoxia and creating a more permissive environment for immune cell trafficking. Furthermore, Tian et al. (68) found that the combination of targeted drugs and PD-1 inhibitors promoted high endothelial venules formation by activating VEGFC and non-canonical NF-κB pathways, thereby increasing the infiltration of CCR7^+^CD8^+^ T cells into HCC. Notably, the higher the proportion of these newly recruited CCR7^+^CD8^+^ T cells within the tumor, the more effectively the accompanying immunotherapy appeared to exert its antitumor effects. Additionally, using subcutaneous HCC mouse models, Jiang et al. (69) found that the combination of Len and anti-PD-1 generated activated immune profiles, characterized by higher proportions of CD4^+^ and CD8^+^ T cells and B cells, along with a lower frequency of MDSCs compared to either Len or anti-PD-1 monotherapy, indicating broad relief of immunosuppression. Beyond modulating immune cell infiltration, targeted therapy can directly enhance tumor immunogenicity. For instance, Sor induces hypoxia, activating the HIF-1α pathway and upregulating PD-L1, whereas Len promotes PD-L1 via the MCT1/NF-κB/COX-2 pathway in HCC cells (70). Building on mechanistic insights, there is a strong rationale for adopting triple therapy that combines TACE with anti-VEGF/TKIs and PD-1/PD-L1 inhibitors. Our analysis encompassed multiple ICIs, involving Ate, Cam, Dur, Pem, Tis, Sin, and Tor. Tis, a novel anti-PD-1 monoclonal antibody, has shown promise in recent trials. A Phase III RCT (71) suggested that Tis could obtain a noninferior benefit compared to Sor for uHCC, indicated by a median OS (mOS) of 15.9 months in the Tis arm and 14.1 months in the Sor arm. Cam is another PD-1 inhibitor widely used in China. A phase I/II trial (72) (NCT02942329) showed that the Apa-Cam combination demonstrated encouraging outcomes in patients with advanced HCC. In addition, Ahmad et al. (73) compared Cam monotherapy with its combinations with other agents and found that among all treatment arms, Len-Cam was associated with the highest OS and PFS. Consistent with these basic research and clinical research lines of evidence, our study demonstrated that triple therapy improved OS and PFS compared with TACE alone, TACE plus targeted drugs, or targeted drugs plus ICIs.

Accumulating evidence from recent studies has shown differences in the efficacy of various triple regimens. Kudo et al. (5) suggested the combination of TACE-Len-Pem may yield superior outcomes, while others (18, 19, 39) paid more attention to the combination of TACE-Len-Tis. In our analysis, five triple regimens were evaluated in patients with uHCC: TACE-Apa-Cam, TACE-Bev-Ate, TACE-Len-Cam, TACE-Len-Pem, and TACE-Len-Tis. Among these combinations, we found that the TACE-Len-Tis combination and the TACE-Bev-Ate regimen appeared to confer greater benefit.

Multiple factors may influence the differential efficacy observed across regimens. From one perspective, the efficacy of specific combinations can be understood in terms of the drugs’ intrinsic characteristics. For instance, Bev and Len exhibited distinct mechanistic profiles that may influence their efficacy when paired with specific PD-1/PD-L1 inhibitors. For Bev, Zhu et al. (74) found that Bev might synergize with Ate through targeting angiogenesis, regulatory T cell proliferation, and myeloid cell inflammation. Beyond the mechanistic evidence, the safety profiles of Bev and Len also warrant consideration. When Bev was combined with Ate, treatment-related grade 5 adverse events (AEs) were observed in only 2% of patients; treatment-related grade 3 or 4 AEs occurred in 43% of patients (4). Similarly, the Len-based triple therapy had no grade 5 AEs; grade 3 or 4 AEs occurred in 33.8% of patients, and only 16.2% discontinued treatment due to treatment-related AEs (18). In our analysis, three Len-based triple therapies were evaluated. Among the three regimens combined with Tis, Cam, and Pem, respectively, TACE-Len-Tis may achieve the most encouraging outcomes, which may be attributed to the characteristics of the PD-1/PD-L1 inhibitors and to the interaction between PD-1/PD-L1 inhibitors and Len. Regarding drugs’ inherent properties, first of all, Pem binds to the flexible C’D loop of PD-1 (75). In contrast, Cam interacts with PD-1 via N-glycosylation at asparagine 58 (76), thereby substantially increasing its binding affinity. Unlike the binding site of Pem and Cam, Tis binds to a critical epitope on PD-1 (Gln75, Thr76, Asp77, and Arg86). More importantly, Tis exhibits approximately a 100-fold slower dissociation rate (Kd) from PD-1 than Pem, resulting in more sustained receptor occupancy and longer PD-1/PD-L1 blockade (77). Additionally, compared with Pem, Tis’s Fc region was engineered to prevent interaction with FcγRs, thereby minimizing antibody-dependent cellular phagocytosis (ADCP) and enhancing T-cell immunity to target and eliminate tumor cells (78). These molecular features, including a unique binding epitope, an extremely low Kd, and an Fc-optimized design, may collectively contribute to the favorable outcomes observed with Tis-based combinations in our analysis.

Beyond the mechanisms of the therapeutic agents involved, drug interactions cannot be ignored and may influence the efficacy of triple therapy. In the CHANCE2211 study (79), the safety profile of the Apa-Cam combination was well documented, with grade 3 or 4 AEs occurring in only 16.7% of patients receiving the triple regimen and no treatment-related deaths. Discontinuation rates were similarly low: 4.8% for Cam and 11.9% for Apa, indicating that the combination was well tolerated and supported long-term treatment adherence. Furthermore, both agents are manufactured by the same Chinese pharmaceutical company (Jiangsu Hengrui Pharmaceuticals), which may facilitate optimized dosing schedules and better compatibility in clinical practice. Several clinical trials have investigated the efficacy of the Apa-Cam combination. For example, a real-world study (80) reported an objective response rate of 35.9%, a disease control rate of 82.1%, and a median PFS of 24 months in patients with resectable HCC. As for the Len-Tis combination, synergistic potential may be inferred from their complementary mechanistic profiles and emerging clinical evidence. Chen et al. (81) found that adding Tis and Len after curative hepatectomy improved disease-free survival to 40.78 months (HR = 0.51) and mOS to 42.1 months (HR = 0.36), compared to 28.8 and 34 months with hepatectomy alone. Mechanistically, Len exerts anti-angiogenic effects by blocking VEGFR1-3, FGFR, and other receptor tyrosine kinases, and has been shown to modulate the immune microenvironment by reducing MDSCs. As for Tis, with its engineered Fc region, it avoids ADCP, a process that can inadvertently eliminate activated T cells, thereby preserving the effector T cell pool. Together, these two agents create a favorable scenario; Len remodels the TME to facilitate immune cell infiltration, while Tis prevents premature depletion of infiltrating T cells. Consistent with these studies, our analysis confirmed the favorable outcomes of the TACE-Len-Tis, TACE-Bev-Ate, and TACE-Apa-Cam regimens. Although the precise mechanisms underlying the synergy of these combinations have yet to be fully clarified, available preclinical and clinical data collectively suggest that the Len-Tis, Bev-Ate, and Apa-Cam combinations may act synergistically to enhance antitumor activity.

In addition, differences between studies warrant attention. Regarding the inclusion criteria of studies, three regimens reported the BCLC stage of enrolled patients: TACE-Apa-Cam, TACE-Len-Cam, and TACE-Len-Pem. The pooled data showed that patients with more advanced disease appeared to derive less benefit. The proportion of patients with BCLC stage C in the experimental groups was 20.27%, 20.47%, and 41.27%, respectively, which corresponded to the PFS HR for the three regimens, 0.45, 0.49, and 0.60. Patients with BCLC stage C disease typically have more comorbidities, are in poorer physical condition, and have worse liver function compared with those with stage A or B disease. Therefore, in regimens that enrolled a higher proportion of BCLC stage C patients, such as TACE-Len-Pem, the pooled PFS was correspondingly lower, not because the regimen itself was less effective, but because the patient population was inherently more fragile. Beyond tumor stage, various factors, including tumor burden, serum alpha-fetoprotein levels, HBV infection status, and others, may help explain the heterogeneity in treatment outcomes observed across studies. However, due to the limited data, we were unable to analyze these factors in our study.

The optimal sequence of TACE and systemic therapies for uHCC remains an area of ongoing research. In our analysis, we observed that both TACE as a pre-treatment strategy and systemic therapy as the first strategy could achieve favorable outcomes. However, given the limited sample size in the latter group, these findings should be interpreted with caution, as they may be influenced by chance. Zheng et al. (82) specifically compared the sequencing of TACE and systemic therapies in 194 patients with BCLC stage C HCC, reporting that the order of initiation did not significantly impact OS and PFS (HR = 0.55, p=0.091 for OS; HR = 1.17, p=0.336 for PFS). These findings suggested that the sequence of TACE and systemic therapy may be less crucial than previously assumed, allowing for greater flexibility in treatment planning based on patient-specific factors.

Notably, current evidence on triple therapy has primarily focused on its first-line use. In our study, most included studies evaluated triple therapy as initial treatment for uHCC. In contrast, only 3 studies were included in the non-first-line therapy group, highlighting a significant evidence gap and suggesting that the efficacy of triple therapy in the non-first-line setting remained largely unexplored. Hwang et al. (83) discussed the current optimal strategies for second-line treatment after the failure of first-line PD-1/PD-L1 inhibitor-based therapy. Nonetheless, in the absence of phase III RCT data, their review could not address the role of triple therapy as a non-first-line treatment, further highlighting the evidence gap. Therefore, well-designed RCTs are urgently needed to determine the efficacy of triple therapy in later lines of treatment.

This study had several strengths worth highlighting. First, this comprehensive, systematic meta-analysis evaluated the efficacy of triple therapy in patients with uHCC. Second, we conducted detailed subgroup analyses from multiple perspectives, including subgroups defined by control regimens, experimental combinations, the sequencing of TACE and systemic therapy, and whether triple therapy was administered as first-line or non-first-line treatment. The results of these analyses provided valuable insights into the optimal triple-therapy regimen across different clinical settings.

Nevertheless, despite the survival benefits of intensive therapies demonstrated in recent meta-analyses of HCC and other solid tumors (84), several limitations of this meta-analysis should be acknowledged. First, large-scale RCTs on this topic remain scarce, with the majority of included studies being cohort studies. This introduces inherent biases, including publication bias, selection bias, performance bias, and detection bias, that may have led to deviations in the estimated true treatment effects. Although we performed sensitivity analyses to assess the robustness of our findings, residual confounding cannot be entirely ruled out. Second, the comparisons made in our analysis were not based on head-to-head trials. The efficacy estimates were derived from indirect comparisons across studies with different designs, patient populations, and treatment protocols, which may have limited the precision of our conclusions. Third, it was worth noting that most of the studies included in our analysis were conducted in China, which may limit the generalizability of our findings to other ethnic groups. In China, the epidemiological profile of HCC was characterized by a high prevalence of HBV infection, accounting for 84.4% of HCC cases (85). Fourth, because few studies are available for certain subgroup analyses, some observed trends may be unstable and warrant confirmation in future research. Fifth, TACE procedures themselves were an additional source of heterogeneity, as the chemotherapeutic agents, embolic materials, and the level of vascular embolization varied across studies. These technical details were not uniformly reported, so a detailed subgroup analysis based on these factors could not be performed. Finally, using the GRADE framework, the overall certainty of the evidence was rated as moderate for both OS and PFS. This rating reflects a balance between the inherent limitations of the observational evidence base and the consistently large effect size observed across studies.

Several ongoing or recently reported phase III trials are poised to further refine the landscape of triple therapy. For example, TALENTACE, a phase III open-label RCT, showed that the TACE-Bev-Ate group achieved significantly improved PFS compared with the TACE-alone group in treatment-naïve uHCC patients with intermediate-to-high tumor burden (86). Nevertheless, important unanswered questions remain, including the optimal sequencing and duration of therapy, as well as the management of cumulative toxicities. More critically, identifying which patient subsets derive the greatest benefit remains a key priority.

## Conclusion

In conclusion, this updated meta-analysis confirmed the efficacy of TACE combined with anti-VEGF/TKIs and PD-1/PD-L1 inhibitors in patients with uHCC. Beyond simply confirming overall efficacy, our detailed analyses provided practical guidance on the optimal use of triple therapy across different clinical settings, which may inform treatment decisions for patients with uHCC.

### Summary box

This meta-analysis of 53 studies involving over 10, 000 patients demonstrated that triple therapy combining transarterial chemoembolization (TACE) with anti-vascular endothelial growth factor/tyrosine kinase inhibitors and programmed cell death protein 1/programmed cell death protein ligand 1 inhibitors significantly improved overall survival and progression-free survival in patients with unresectable hepatocellular carcinoma compared with other treatment regimens. Among the evaluated combinations, TACE plus bevacizumab and atezolizumab, and TACE plus lenvatinib and tislelizumab, seem to offer greater benefits. The survival advantage of triple therapy was observed regardless of whether TACE or systemic therapy was initiated first, and in both first-line and non-first-line settings. These findings support triple therapy as an effective treatment strategy.

## Data Availability

The datasets presented in this study can be found in online repositories. The names of the repository/repositories and accession number(s) can be found in the article/**Supplementary Material**.

## Supplementary material

Supplementary Figure 1 Risk of bias assessment in RCT studies.

Supplementary Figure 2 Summary of risk of bias across RCT studies.

Supplementary Figure 3 Egger’s test for OS in the meta-analysis of PD-1/PD-L1 inhibitors combined with anti-VEGF/TKIs and TACE in uHCC (p=0.056).

Supplementary Figure 4 Plot of the sensitivity analysis regarding the OS for the PD-1/PD-L1 inhibitors combined with anti-VEGF/TKIs and TACE in uHCC.

Supplementary Figure 5 Egger’s test for PFS in the meta-analysis of PD-1/PD-L1 inhibitors combined with anti-VEGF/TKIs and TACE in uHCC (p=0.112).

Supplementary Figure 6 Plot of the sensitivity analysis regarding the PFS for the PD-1/PD-L1 inhibitors combined with anti-VEGF/TKIs and TACE in uHCC.
