# Supplementary material for: Efficacy of PD-1/PD-L1 inhibitors combined with anti-VEGF/TKIs and TACE in uHCC: a meta-analysis
Source: Front Oncol. 2026 Sep 14;16:1927223. doi: 10.3389/fonc.2026.1927223 (PMC13616637; doi:10.3389/fonc.2026.1927223)
Supplement: Supplementary file 1 [file Table1.docx]

**Supplementary Table 1. Characteristics of the studies included in this meta-analysis.**

| Author | Year | Recruitment time | Experimental group | | | Control group | | |
| --- | --- | --- | --- | --- | --- | --- | --- | --- |
|  |  |  | ECOG PS, n  0/1/2 | BCLC stage, n  A/B/C | Child-Pugh class, n  A/B | ECOG PS, n  0/1/2 | BCLC stage, n  A/B/C | Child-Pugh class, n  A/B |
| Kudo M | 2025 | 2020.05-2023.01 | 216/21/0 | 80/135/21 | 237/0 | 213/30/0 | 68/146/29 | 243/0 |
| Sangro B | 2025 | 2018.11-2021.07 | 167/37/0 | 51/117/35 | 200/4 | 175/30/0 | 49/122/31 | 201/4 |
| Zhu HD | 2026 | 2020.12-2023.10 | 56/44/0 | 14/44/42 | 100/0 | 58/42/0 | 14/42/44 | 100/0 |
| Yang D-L | 2025 | 2019.01-2023.06 | NR | 56/95/150 | 245/56 | NR | 59/97/145 | 237/64 |
| Yao Y | 2025 | 2019.01-2023.04 | NR | 0/39/0 | 34/5 | NR | 0/39/0 | 34/5 |
| Wang M-X | 2025 | 2018.12-2023.01 | 29/93/0 | 0/83/39 | 81/41 | 17/35/0 | 0/33/19 | 35/17 |
| Zheng Y | 2024 | 2021.06-2022.03 | NR | 0/42/0 | 34/8 | NR | 0/42/0 | 36/6 |
| Guo C | 2025 | 2019.02-2023.03 | NR | 0/8/18 | 22/4 | NR | 0/9/17 | 24/2 |
| Yuan G | 2024 | 2016.04-2021.12 | 118/21/0 | 0/99/40 | 119/20 | 233/45/0 | 0/196/82 | 237/41 |
| Tang Z | 2024 | 2013.09-2023.06 | 49/21/0 | 13/27/30 | 62/8 | 50/20/0 | 15/27/28 | 59/11 |
| Jin ZC | 2024 | 2018.01-2022.12 | 464/308/33 | NR | 659/146 | 257/162/18 | NR | 357/80 |
| Jiang X | 2024 | 2019.06-2022.11 | NR | 0/8/36 | 31/13 | NR | 0/11/72 | 67/16 |
| Jiang J | 2024 | 2021.01-2023.06 | 54/14/0 | 0/23/45 | 61/7 | 53/15/0 | 0/22/46 | 59/9 |
| Chen S | 2024 | 2016.01-2022.12 | NR | NR | 59/7 | NR | NR | 72/11 |
| Cao L | 2025 | 2019.03-2022.06 | 26/30/0 | 10/46/0 | 46/10 | 25/31/0 | 11/45/0 | 46/10 |
| Yang H | 2023 | 2019.01-2022.01 | 12/46/0 | 0/26/38 | 38/26 | 11/53/0 | 0/24/34 | 33/25 |
| Wu J | 2023 | 2020.01-2021.11 | 0/40/17 | 0/0/57 | 19115 | 0/56/28 | 0/0/84 | 27638 |
| Wang J | 2023 | 2019.11-2021.11 | 16/27/0 | 0/8/35 | 12997 | 14/29/0 | 0/7/36 | 12328 |
| Sun T | 2023 | 2018.01-2020.10 | 32/21/0 | NR | 16316 | 33/20/0 | NR | 15980 |
| Li H | 2023 | 2021.07-2022.07 | 79/87/0 | 0/39/127 | 129/37 | 66/91/0 | 0/28/129 | 123/34 |
| Lang M | 2023 | 2018.12-2020.10 | 48/24/3 | 0/32/43 | 59/16 | 46968 | 0/14/25 | 46295 |
| Gao Y | 2023 | 2019.01-2022.01 | 48/9/0 | 0/17/40 | 34/23 | 40/10/0 | 0/21/29 | 33/17 |
| Duan X | 2023 | 2019.01-2021.06 | 262/187/0 | 0/78/371 | 175/274 | 256/193/0 | 0/75/374 | 185/264 |
| Zhu D | 2022 | 2018.01-2022.01 | 19/15/0 | 0/13/21 | 46142 | 34/34/0 | 0/26/42 | 20790 |
| Xiang YJ | 2022 | 2019.01-2020.01 | NR | 0/56/0 | 56/0 | NR | 0/47/0 | 47/0 |
| Qu S | 2022 | 2017.11-2020.09 | 49/7/0 | 0/17/39 | 19419 | 45/9/0 | 0/18/36 | 18688 |
| Zou X | 2023 | 2018.02-2022.05 | 17/53/0 | 0/0/70 | 46/24 | 28/62/0 | 0/0/90 | 61/29 |
| Hong X | 2024 | 2019.01-2023.06 | 40/37/0 | 0/0/77 | 58/19 | 38/24/0 | 0/0/62 | 40/22 |
| Gao B | 2023 | 2019.01-2022.12 | NR | 36797 | 46293 | NR | 36856 | 46263 |
| Cao F | 2023 | 2021.04-2022.10 | 30/32/0 | NR | 40/22 | 32/45/0 | NR | 51/26 |
| Li H | 2023 | 2018.10-2022.12 | NR | 3/5/37 | 13058 | NR | 4/6/35 | 32/13 |
| Sheng Y | 2024 | 2016.01-2021.12 | 62/36/15 | 0/54/59 | 87/26 | 66/42/20 | 0/64/64 | 100 /28 |
| Jin M | 2024 | 2020.08-2023.09 | 29/10/0 | 0/19/20 | 46295 | 31/8/0 | 0/17/22 | 46295 |
| Zhao Y | 2024 | 2021.03-2023.09 | NR | 0/17/86 | 90/19 | NR | 0/11/55 | 21125 |
| Xia WL | 2022 | 2018.12-2021.06 | 12/47/0 | 0/0/59 | 20515 | 12/47/0 | 0/0/59 | 20852 |
| Chen S | 2022 | 2016.07-2020.07 | 27/43/0 | 0/47/23 | 70/0 | 30/42/0 | 0/45/27 | 72/0 |
| Pan H | 2025 | 2016.01-2021.12 | 29/31/0 | 9/18/33 | 19207 | 30/30/0 | 7/11/42 | 19207 |
| Han Z | 2022 | 2016.06-2021.10 | NR/NR/20 | NR/NR/17 | 14550 | NR/NR/38 | NR/NR/42 | 81/40 |
| Xue M | 2025 | 2015.01-2023.12 | 22/13/0 | NR | NR | 53/27/0 | NR | NR |
| Liu S | 2025 | 2018.01-2023.12 | NR | 0/15/27 | 20/22 | NR | 0/18/24 | 26/16 |
| Liu C-Y | 2025 | 2018.01-2024.06 | 24/30/0 | 0/0/54 | 16346 | 26/31/0 | 0/0/57 | 17777 |
| Lei Y | 2025 | 2021.01-2024.10 | 78/129/3 | 4/29/177 | 159/51 | 31/43/2 | 4/14/58 | 54/22 |
| Zhang N | 2025 | 2020.07-2023.12 | 59/99/0 | 0/42/117 | 89/69 | 55/100/0 | 0/44/111 | 95/60 |
| Zhu R | 2025 | 2021.01-2024.12 | 48/35/0 | 0/0/83 | NR | 43/41/0 | 0/0/84 | NR |
| Zhang Q | 2026 | 2021.01-2024.01 | 20/38/0 | 0/37/21 | 32/26 | 16/42/0 | 0/31/27 | 34/24 |
| Yang ZR | 2025 | 2020.05-2024.05 | 59/40/26 | 0/0/134 | NR | 46/42/25 | 0/0/126 | NR |
| Wang D | 2026 | 2020.02-2024.07 | 8/38/0 | 0/11/35 | 23/23 | 8/38/0 | 0/8/38 | 22/24 |
| Chen JJ | 2026 | 2018.01-2022.12 | NR | 0/307/0 | 264/43 | NR | 0/633/0 | 545/88 |
| Zhao H | 2026 | 2016.06-2022.12 | NR/NR/25 | 0/41/72 | 89/24 | NR/NR/24 | 0/32/64 | 74/22 |
| Liang Y | 2025 | 2020.01-2024.01 | NR | NR | NR | NR | NR | NR |
| Li Z | 2026 | 2019.02-2022.08 | NR | 0/13/28 | 37/4 | NR | 0/46/55 | 68/33 |
| Hashimoto K | 2025 | 2020.10-2024.04 | NR | 7/29/13 | 47/2 | NR | 6/28/15 | 49/0 |
| Xu HW | 2025 | 2020.01-2023.12 | NR | 0/22/83 | 64/41 | NR | 0/25/80 | 70/35 |

ECOG PS, Eastern Cooperative Oncology Group performance status; BCLC, Barcelona Clinic Liver Cancer; NR, not reported.
