# Supplementary material for: Efficacy of PD-1/PD-L1 inhibitors combined with anti-VEGF/TKIs and TACE in uHCC: a meta-analysis
Source: Front Oncol. 2026 Sep 14;16:1927223. doi: 10.3389/fonc.2026.1927223 (PMC13616637; doi:10.3389/fonc.2026.1927223)
Supplement: Supplementary file 2 [file Table3.docx]

**Supplementary Table 3. GRADE evidence profile for triple therapy versus non‑triple therapy in unresectable hepatocellular carcinoma.**

Generated in accordance with GRADEpro methodology. The evidence base comprised 3 randomised controlled trials and 50 cohort studies; starting quality was rated as LOW due to the predominance of observational studies.

| **Certainty assessment** | | | | | | | | **№ of patients** | | **Effect** | | **Certainty** | **Importance** |
| --- | --- | --- | --- | --- | --- | --- | --- | --- | --- | --- | --- | --- | --- |
| **Outcomes** | **№ of studies** | **Study design** | **Risk of bias** | **Inconsistency** | **Indirectness** | **Imprecision** | **Other considerations** | **Triple Therapy** | **Non-triple therapy** | **Relative (95% CI)** | **Absolute (95% CI)** |  |  |
| *Overall Survival* | 51 | non-randomised studies | serious^a^ | serious^b^ | not serious | not serious | strong association | 5514 participants | 5316 participants | **HR 0.47** (0.43 to 0.53) [OS] | **222 fewer per 1,000** (from 242 fewer to 193 fewer) | ⨁⨁⨁◯ Moderate^a,b^ | CRITICAL |
|  |  |  |  |  |  |  |  | - | 50.0% |  | **222 fewer per 1,000** (from 242 fewer to 193 fewer) |  |  |
| *Progression-Free Survival* | 49 | non-randomised studies | serious^c^ | serious^d^ | not serious | not serious | strong association | 5511 participants | 5135 participants | **HR 0.52** (0.47 to 0.57) [PFS] | **229 fewer per 1,000** (from 261 fewer to 200 fewer) | ⨁⨁⨁◯ Moderate^c,d^ | IMPORTANT |
|  |  |  |  |  |  |  |  | - | 65.0% |  | **229 fewer per 1,000** (from 261 fewer to 200 fewer) |  |  |

GRADE, Grading of Recommendations Assessment, Development and Evaluation; CI, confidence interval; HR, hazard ratio.

Explanalions:

a. Most evidence derived from cohort studies with inherent confounding and selection bias, despite adjustment in original analyses.

b. I² > 50% in most subgroups, indicating substantial heterogeneity.

c. Predominantly cohort-based evidence; inherent risk of confounding and selection bias.

d. I² > 50% across multiple subgroups, indicating heterogeneity.
