# Supplementary material for: Efficacy of PD-1/PD-L1 inhibitors combined with anti-VEGF/TKIs and TACE in uHCC: a meta-analysis
Source: Front Oncol. 2026 Sep 14;16:1927223. doi: 10.3389/fonc.2026.1927223 (PMC13616637; doi:10.3389/fonc.2026.1927223)
Supplement: Supplementary file 3 [file Table2.doc]

**Supplementary Table 2. Quality assessment of the** cohort studies included.

| Author, year | **Selection (Out of 4)** | | | | **Comparability**  **(Out of 2)** | **Outcomes (Out of 3)** | | | **Total**  **(Out of 9)** |
| --- | --- | --- | --- | --- | --- | --- | --- | --- | --- |
|  | Representativeness of exposed cohort | Selection of non exposed cohort | Ascertainment  of exposure | Outcome not present at the start of the study |  | Assessment of outcomes | Length of follow-up | Adequacy of follow up of cohorts |  |
| Yang D-L, 2025 | 1 | 1 | 1 | 1 | 2 | 1 | 1 | 0 | 8 |
| Yao Y, 2025 | 1 | 1 | 1 | 1 | 2 | 1 | 1 | 0 | 8 |
| Wang M-X, 2025 | 1 | 1 | 1 | 1 | 1 | 1 | 1 | 0 | 7 |
| Zheng Y, 2024 | 1 | 1 | 1 | 1 | 2 | 1 | 1 | 0 | 8 |
| Guo C, 2025 | 1 | 1 | 1 | 1 | 2 | 1 | 1 | 0 | 8 |
| Yuan G, 2024 | 1 | 1 | 1 | 1 | 2 | 1 | 1 | 0 | 8 |
| Tang Z, 2024 | 1 | 1 | 1 | 1 | 2 | 1 | 0 | 0 | 7 |
| Jin ZC, 2024 | 1 | 1 | 1 | 1 | 1 | 1 | 1 | 0 | 7 |
| Jiang X, 2024 | 1 | 1 | 1 | 1 | 1 | 1 | 1 | 0 | 7 |
| Jiang J, 2024 | 1 | 1 | 1 | 1 | 1 | 1 | 0 | 0 | 6 |
| Chen S, 2024 | 1 | 1 | 1 | 1 | 1 | 1 | 1 | 0 | 7 |
| Cao L, 2025 | 1 | 1 | 1 | 1 | 1 | 1 | 0 | 0 | 6 |
| Yang H, 2023 | 1 | 1 | 1 | 1 | 1 | 1 | 1 | 0 | 7 |
| Wu J, 2023 | 1 | 1 | 1 | 1 | 1 | 1 | 0 | 0 | 6 |
| Wang J, 2023 | 1 | 1 | 1 | 1 | 2 | 1 | 1 | 0 | 8 |
| Sun T, 2023 | 1 | 1 | 1 | 1 | 2 | 1 | 0 | 1 | 8 |
| Li H, 2023 | 1 | 1 | 1 | 1 | 1 | 1 | 0 | 0 | 6 |
| Lang M, 2023 | 1 | 1 | 1 | 1 | 2 | 1 | 0 | 0 | 7 |
| Gao Y, 2023 | 1 | 1 | 1 | 1 | 1 | 1 | 1 | 0 | 7 |
| Duan X, 2023 | 1 | 1 | 1 | 1 | 2 | 1 | 1 | 0 | 8 |
| Zhu D, 2022 | 1 | 1 | 1 | 1 | 1 | 1 | 1 | 0 | 7 |
| Xiang YJ, 2022 | 1 | 1 | 1 | 1 | 1 | 1 | 0 | 0 | 6 |
| Qu S, 2022 | 1 | 1 | 1 | 1 | 1 | 1 | 1 | 0 | 7 |
| Zou X, 2023 | 1 | 1 | 1 | 1 | 1 | 1 | 1 | 1 | 8 |
| Hong X, 2024 | 1 | 1 | 1 | 1 | 0 | 1 | 1 | 1 | 7 |
| Gao B, 2023 | 1 | 1 | 1 | 1 | 2 | 1 | 0 | 0 | 7 |
| Cao F, 2023 | 1 | 1 | 1 | 1 | 1 | 1 | 0 | 0 | 6 |
| Li H, 2023 | 1 | 1 | 1 | 1 | 2 | 1 | 1 | 1 | 9 |
| Sheng Y, 2024 | 1 | 1 | 1 | 1 | 2 | 1 | 1 | 0 | 8 |
| Jin M, 2024 | 1 | 1 | 1 | 1 | 2 | 1 | 1 | 0 | 8 |
| Zhao Y, 2024 | 1 | 1 | 1 | 1 | 1 | 1 | 1 | 1 | 8 |
| Xia WL, 2022 | 1 | 1 | 1 | 1 | 2 | 1 | 1 | 0 | 8 |
| Chen S, 2022 | 1 | 1 | 1 | 1 | 0 | 1 | 1 | 0 | 6 |
| Pan H, 2025 | 1 | 1 | 1 | 1 | 2 | 1 | 1 | 1 | 9 |
| Han Z, 2022 | 1 | 1 | 1 | 1 | 1 | 1 | 1 | 0 | 7 |
| Xue M, 2025 | 1 | 1 | 1 | 1 | 1 | 1 | 1 | 0 | 7 |
| Liu S, 2025 | 1 | 1 | 1 | 1 | 2 | 1 | 0 | 1 | 8 |
| Liu C-Y, 2025 | 1 | 1 | 1 | 1 | 0 | 1 | 1 | 0 | 6 |
| Lei Y, 2025 | 1 | 1 | 1 | 1 | 1 | 1 | 1 | 0 | 7 |
| Zhang N, 2025 | 1 | 1 | 1 | 1 | 2 | 1 | 1 | 0 | 8 |
| Zhu R, 2025 | 1 | 1 | 1 | 1 | 1 | 1 | 0 | 1 | 7 |
| Zhang Q, 2026 | 1 | 1 | 1 | 1 | 1 | 1 | 1 | 1 | 8 |
| Yang ZR, 2025 | 1 | 1 | 1 | 1 | 0 | 1 | 1 | 0 | 6 |
| Wang D, 2026 | 1 | 1 | 1 | 1 | 2 | 1 | 1 | 1 | 9 |
| Chen JJ, 2026 | 1 | 1 | 1 | 1 | 1 | 1 | 1 | 1 | 8 |
| Zhao H, 2026 | 1 | 1 | 1 | 1 | 1 | 1 | 1 | 0 | 7 |
| Liang Y, 2025 | 1 | 1 | 1 | 1 | 2 | 1 | 1 | 1 | 9 |
| Li Z, 2026 | 1 | 1 | 1 | 1 | 0 | 1 | 1 | 1 | 7 |
| Hashimoto K, 2025 | 1 | 1 | 1 | 1 | 2 | 1 | 1 | 0 | 8 |
| Xu HW, 2025 | 1 | 1 | 1 | 1 | 0 | 1 | 1 | 0 | 6 |

The cohort studies were assessed by the Newcastle-Ottawa Quality Assessment Scale (NOS) checklist.
